# Association between digital smart device use and depression among older adults: systematic review and meta-analysis

**DOI:** 10.1186/s12889-026-27433-x

**Published:** 2026-05-09

**Authors:** Juan Gu, Xiaohong Zhang, Yake Yue, Mengjie Tong, Yufei Qiu, Jiali Liu, Yong Zhou, Fen Yang

**Affiliations:** 1 Hubei University of Chinese Medicine, Wuhan, 430065 China; 2 Affiliated Hospital of Hubei University of Chinese Medicine, 430061 Wuhan, China; 3 Hubei Shizhen Laboratory, 430065 Wuhan, China; 4 Hubei Province Academy of Traditional Chinese Medicine, Wuhan, 430061 China; 5 Hubei Key Laboratory of theory and application research of liver and kidney in traditional Chinese medicine, Affiliated Hospital of Hubei University of Chinese Medicine, 430061 Wuhan, China; 6 Department of Geriatrics, Hubei Provincial Hospital of Traditional Chinese Medicine, 430061 Wuhan, China

**Keywords:** Digital smart device use, Depression, Older adults, Meta-analysis, Systematic review

## Abstract

**Background:**

Depression among older adults is increasingly emerging as a global public health concern. With the rapid advancement of digital information technology, digital smart devices have profoundly reshaped the lifestyle of older adults and may influence their mental health. However, the association between digital smart device use and depression in this population remains unclear, with existing studies reporting inconsistent findings. This study employed a three-level meta-analytic approach to systematically explore the association between digital smart device use and depression among older adults.

**Methods:**

Systematic searches were conducted on six electronic databases including PubMed, Cochrane Library, Embase, Web of Science, MEDLINE, and PsycINFO from their inception to May 6, 2025. Original observational studies (cross-sectional or cohort) were included. Exclusion criteria comprised non-English publications, review articles, conference papers or abstracts, duplicate publications, and studies without available full text or required data. Two researchers independently performed literature screening, data extraction, and quality assessment. Three-level random-effects meta-analyses were conducted to estimate the pooled standardized beta coefficients (*β*) for continuous depression outcomes and odds ratios (OR) for binary depression outcomes. Risk of bias was assessed using the Newcastle–Ottawa Scale (NOS) for cohort studies and the AHRQ criteria for cross-sectional studies. Potential moderators were explored using three-level meta-regression, and potential publication bias was evaluated using funnel plots, modified Egger’s test, and trim-and-fill analysis.

**Results:**

A total of 28 articles (23 cross-sectional, 5 cohort) published between 2014 and 2025 were included in the systematic review, with 24 eligible for meta-analysis. All included studies were deemed high quality (mean NOS score: 7.60; mean AHRQ score: 7.57). For continuous outcomes, digital smart device use was significantly associated with lower depression scores (*β* = -0.201, 95% *CI*: -0.324, -0.079, *p* < 0.01). For binary outcomes, device use was associated with a decreased risk of depression (OR = 0.676, 95% *CI*: 0.613, 0.745, *p* < 0.001). Moderator analyses indicated that the association was not significantly modified by age, study design, sample size, region, measurement tools, or publication year (*p* > 0.05).

**Conclusions:**

This study has certain limitations, including publication bias (*p* < 0.001). However, trim-and-fill analyses confirmed that the results remained statistically significant after imputing missing studies. This three-level meta-analysis provides robust evidence that digital device use is inversely associated with depression among older adults, supporting their potential role in mental health promotion.

**Trial registration:**

The study protocol was registered with the International Prospective Register of Systematic Reviews (PROSPERO) in 2024 (registration number: CRD42024616432).

**Supplementary Information:**

The online version contains supplementary material available at 10.1186/s12889-026-27433-x.

## Introduction

Depression is a prevalent mental health disorder characterized by emotional dysregulation, leading to persistent sadness, loss of interest, and anhedonia [1–3]. With the increasing aging population worldwide, the prevalence of depression among older adults is rising, posing a significant challenge to public health [4–6]. Estimates suggest that the prevalence of clinical symptoms of depression ranges from 10 to 15% among adults aged 65 and above [7]. A recent meta-analysis reported a depression prevalence of 31.7% (95% CI: 27.9–35.6) in older adults, with higher rates observed in those with somatic comorbidities [8]. Depression in older adults significantly impairs their physical health by increasing the risk of cardiovascular diseases, cognitive decline, and sleep disorders [9, 10]. It also negatively affects their psychological well-being through reduced quality of life, social isolation, and increased risk of suicide [11, 12]. Therefore, it is essential to identify modifiable factors influencing depression in this vulnerable population to develop effective interventions and improve their mental well-being.

With the rapid development of digital information technology, an increasing number of older adults are adopting digital smart devices, including smartphones, computers, and tablets [13]. Recent studies indicate that over 70% of older adults now use at least one digital smart device [14–18], with usage rates continuing to rise as technology becomes more accessible and user-friendly. Emerging evidence suggests digital smart devices may significantly influence older adults’ mental health through multiple pathways. Firstly, digital smart devices can enhance social connectivity by allowing older adults to maintain regular contact with family and friends through video calls and social media platforms. This increased social interaction can help reduce feelings of loneliness and isolation, which are significant risk factors for depression [19, 20]. Secondly, digital smart devices provide access to a wide range of entertainment and educational content, offering older adults opportunities for mental stimulation and personal growth [21]. For instance, online courses, e-books, and puzzle games can help keep the mind active and engaged. Thirdly, digital smart devices can also serve as tools for cognitive training, potentially delaying cognitive decline and improving overall mental well-being [22, 23]. However, despite these potential benefits, the relationship between digital smart device use and depression in older adults remains unclear. Existing studies have begun to explore this association, but their findings are inconsistent. Some studies have reported a negative correlation between digital smart device use and depression, suggesting that use is associated with lower levels of depressive symptoms [24, 25]. Other studies indicated a positive correlation between digital smart device use and depression [26, 27]. This inconsistency makes it difficult to determine whether digital smart device use is a protective factor or a risk factor for depression among older adults. Moreover, most existing studies on the association between digital smart device use and depression employ cross-sectional designs, lacking systematic reviews or meta-analyses. Therefore, a comprehensive and systematic approach is needed to better understand the complex relationship between digital smart device use and depression among older adults.

This study aimed to explore the association between digital smart device use and depression among older adults through a comprehensive synthesis of existing evidence using a three-level meta-analytic approach. The theoretical implications of this work will advance our understanding of how technology interacts with mental health in aging populations. From a practical standpoint, the results will inform targeted interventions at multiple levels, including individual training programs for older adults, community-based digital literacy initiatives, and policy recommendations for technology integration in geriatric mental health care. Ultimately, this research contributes to the development of evidence-based strategies for leveraging digital technologies to improve mental well-being in our aging society.

## Methods

The study protocol was registered with the International Prospective Register of Systematic Reviews (PROSPERO) under registration number CRD42024616432. The review was conducted in accordance with the Preferred Reporting Items for Systematic Reviews and Meta-Analyses (PRISMA) guidelines [28] and the Meta-analysis of Observational Studies in Epidemiology (MOOSE) [29] guidelines. This study follows the PRISMA statement, as detailed in Supplementary Material 1.

### Search strategies

To identify all potentially relevant articles, a systematic and exhaustive search was performed in six electronic databases: PubMed, Cochrane Library, Embase, Web of Science, MEDLINE, and PsycINFO. The search timeframe spanned from the inception of each database to May 6, 2025, and only publications in the English language were considered. Both free-text keywords and Medical Subject Headings (MeSH) terms were incorporated, using Boolean operators (“AND”, “OR”) to refine and connect search terms. The comprehensive search strategies are detailed in Supplementary Tables S1-S7.

### Selection criteria

The inclusion criteria were as follows: (1) Original observational studies (cross-sectional or cohort designs) with empirical data; (2) Studies reporting the association between digital smart device use and depression using multivariate analysis, controlling for confounders; (3) Studies reporting at least one of the following statistical measures: standardized beta coefficients (*β*), correlation coefficients (*r*), or odds ratios (OR); (4) Studies focusing on older adults, specifically those aged 60 years and older (Studies with participants aged ≥ 45 were included if the mean age was ≥ 60); (5) Digital smart device use was defined as the use of at least one of smartphones, computers, and tablets, or any combination thereof; (6) Studies reporting the sample size; and (7) Use of unique datasets.

The exclusion criteria included: (1) Non-English publications; (2) Reviews, conference papers, or abstracts; (3) Duplicate publications; and (4) Studies for which the full text or required data were unavailable.

### Study selection

The process of literature screening was independently conducted by two researchers (JG and YKY). After merging search results, duplicate records were deleted, followed by a two-stage screening of titles/abstracts followed by full-texts. Articles that clearly did not meet inclusion criteria were excluded at the title/abstract stage. Full-text articles were then reviewed for eligibility. Any disagreements between researchers were resolved through discussion, or when necessary, adjudicated by a third researcher (FY). Data from included studies were extracted into a standardized form, recording information such as author, year, study design, country, sample size, age, gender distribution, and depression measurement tools.

### Quality assessment

Two researchers (JG and YKY) independently evaluated the methodological quality of the included study. The Agency for Healthcare and Research and Quality tool (AHRQ) [30] was employed for cross-sectional studies, comprising 11 items scored as ‘yes’ (1 point), ‘no’, or ‘unclear’ (0 points). The overall score, ranging from 0 to 11, is the sum of all items. For cohort studies, the Newcastle–Ottawa Scale (NOS) [31] was used, which evaluates studies across 8 items with a maximum score of 9. Based on their total scores, studies were categorized as of poor, fair, or good quality. Any discrepancies between researchers were discussed until consensus was achieved or resolved by consulting the third researcher (FY).

### Statistical analysis

All analyses were performed using the metafor package in R software (version 4.4.1).

#### Three-level meta-analytic framework

Traditional meta-analysis methods assume that effect sizes are independent [32]. However, several studies included in this review reported multiple effect sizes derived from the same sample, introducing potential dependency. To rigorously account for this dependency, we adopted a three-level random-effects meta-analysis framework [32]. This model explicitly decomposes the total variance into three levels: sampling variance of observed effect sizes (level 1), within-study variance (level 2), and between-study variance (level 3) [33, 34]. By modeling these nested dependencies, this approach allows for the inclusion of all relevant effect sizes, thereby maximizing information retention and improving statistical efficiency [35].

#### Main analysis and heterogeneity tests

Two separate meta-analyses were conducted: one synthesizing *β* for continuous depression outcomes and another pooling OR for binary outcomes. The restricted maximum likelihood (REML) estimator was used. Heterogeneity was assessed by examining the variance components at level 2 and level 3. Likelihood ratio tests (LRT) were performed to evaluate whether the variance at each level was statistically significant. To explore potential sources of heterogeneity, univariate three-level meta-regression analyses were conducted. Moderators including age, study design, sample size, region, measurement tools, and publication year were tested. Variables showing evidence of moderation or clinical relevant were subsequently considered for multivariable models.

#### Sensitivity analyses

To further evaluate the robustness of the pooled estimates, leave-one-out sensitivity analyses were conducted by sequentially excluding all effect sizes associated with a single study to examine its impact on the overall results [36].

#### Publication bias

Publication bias was assessed using visual inspection of funnel plots and statistical tests. The modified Egger’s regression test was employed, where the standard error was included as a moderator in the three-level model to test for small-study effects while accounting for data dependency [37]. A *p*-value < 0.05 was considered indicative of potential publication bias. In cases where significant asymmetry was detected, the trim-and-fill method was applied to impute potentially missing studies and adjust the pooled estimates [38]. For binary outcomes, the DerSimonian-Laird estimator was used in the trim-and-fill analysis to ensure model convergence.

## Results

### Study selection

**Fig. 1:**
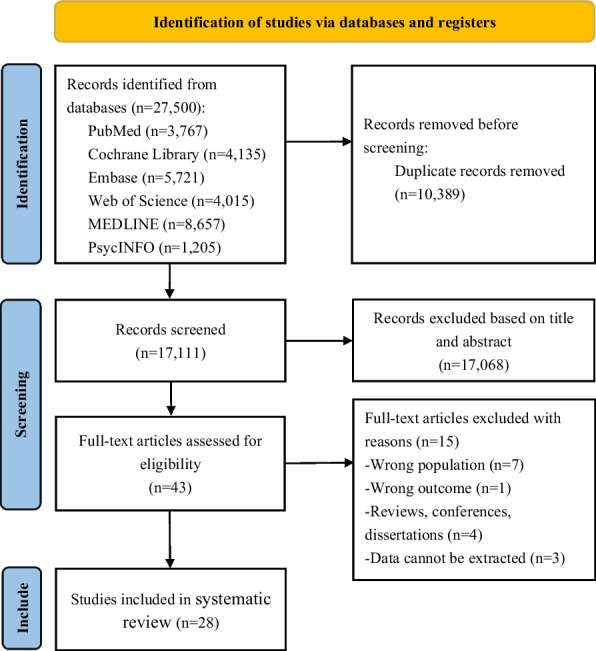
Flow diagram of the literature screening process and result

In our study, a total of 27,500 studies were searched, 10,389 studies were found to be duplicates, and 17,068 were excluded based on title and abstract screening. The remaining 43 studies underwent full-text reading rescreening, 15 were excluded with reasons listed in Supplementary Table S8, resulting in a total of 28 studies included in this systematic review, of which 24 were included in the meta-analysis (Fig. 1).

### Study characteristics

A total of 28 studies were included, and Table 1 summarized the characteristics of these studies. These studies were published between 2014 and 2025, encompassing 264,574 participants across multiple countries. All studies comprised 23 cross-sectional studies and 5 cohort studies. Sample sizes ranged from 235 to 79,702 individuals. For the meta-analysis, 19 studies with 27 effect sizes reported continuous outcomes, while 7 studies with 17 effect sizes provided binary outcomes. It is important to note that 2 studies contributed data to both analyses. Additionally, 4 studies reporting r were included in the systematic review but excluded from meta-analysis due to incompatible data formats.

**Table 1:** Characteristics of the included studies

Author	Publication year	Study design	Country	No.of Participants	Age(y)	Gender, male(%)	Depression measurement	Quality assessment score
Uemura [39]	2018	Cohort	Japan	3,106	≥ 65, 71.5 ± 5.2	50.9	GDS	8
Nakagomi [40]	2022	Cohort	Japan	8,403	≥ 65, 73.0 ± 5.5	45.6	GDS	6
Lin [41]	2020	CS	China	235	≥ 65, 82.6 ± 5.5	41.3	GDS	7
Zhang [42]	2025	CS	China	2,550	≥ 45, 62.0	48.0	CES-D	8
Hwang [43]	2025	CS	Korea	9,920	≥ 65, 73.4 ± 6.5	40.0	SGDS-K	6
Wang [44]	2019	CS	China	7,779	≥ 60, 68.2 ± 6.6	50.0	CES-D	8
Liao [45]	2020	CS	China	18,492	≥ 45, 59.0 ± 9.3	48.6	CES-D	9
Minagawa [46]	2014	Cohort	Japan	5,164	≥ 65, 76.8	43.7	CES-D	8
Ma [47]	2025	CS	China	10,946	≥ 45	49.4	CES-D	7
Chen [14]	2023	CS	China	6,648	≥ 60, 70.9 ± 7.1	49.8	CES-D	9
Ji [48]	2023	CS	China	5,244	≥ 60, 70.7	50.3	CES-D	9
Lee [49]	2021	CS	Korea	10,055	≥ 65, 73.9 ± 6.5	42.5	CES-D	7
Mu [50]	2021	CS	China	8,853	≥ 45, 60.4 ± 10.3	55.5	CES-D	8
Choudhary [51]	2020	CS	China	14,587	≥ 45, 61.0 ± 9.3	48.7	CES-D	9
Xie [52]	2021	CS	China	6,972	≥ 60, 70.3 ± 7.5	51.1	DTS	6
Lu [53]	2024	CS	China	10,654	≥ 60, 68.5 ± 6.2	48.5	CES-D	7
Cotten [54]	2014	Cohort	America	12,300	≥ 50	NA	CES-D	8
Liu [55]	2024	Cohort	China	5,837	≥ 60, 67.1 ± 5.8	51.1	CES-D	8
Ding [15]	2024	CS	China	376	≥ 60, 69.2 ± 11.9	42.8	GDS	8
Kim [56]	2020	CS	America	4,976	≥ 65, 73.7 ± 0.1	45.7	PHQ-2	9
Guo [57]	2025	CS	China	79,702	≥ 45, 60.3 ± 9.7	47.9	CES-D	9
Jiao [58]	2025	CS	China	9,162	≥ 60	50.3	CES-D	7
Guo [59]	2025	CS	China	6,551	≥ 60, 67.7 ± 5.8	51.2	CES-D	8
Nan [60]	2023	CS	China	4,714	≥ 60, 68.1	52.5	CES-D	7
Karaş [27]	2023	CS	NA	392	≥ 65, 65.6 ± 0.9	77.3	GDS	5
Chopik [61]	2016	CS	America	591	≥ 60, 68.2 ± 10.8	55.5	CES-D	8
Koong [25]	2022	CS	China	9,906	≥ 65, 73.7 ± 6.5	40.1	SGDS-K	7
Tian [26]	2023	CS	China	459	≥ 60, 63.4 ± 4.0	43.1	GDS	6

*CS* Cross-sectional study, *NA* Not applicable, *GDS* Geriatric Depression Scale, *CES-D* Center for Epidemiologic Studies Depression Scale, *SGDS-K* Geriatric Depression Scale Short Form Korean Version, *DTS* Depressive Tendency Scale, *PHQ-2* Patient Health Questionnaire-2

The operationalization of digital smart device use varied across the included studies (Supplementary Table S9). While the majority of studies assessed a combination of smartphones, computers, and tablets, others focused exclusively on specific device types. Device use was predominantly operationalized as a binary variable (User vs. Non-user). Definitions typically relied on current ownership, usage within a specific recall period (e.g., past month or year), or device count (e.g., non-users vs. users of ≥ 1 device). A smaller subset of studies incorporated more granular metrics, capturing usage frequency (e.g., “rarely” vs. “regularly”) or daily duration categories. Additionally, several studies characterized usage through engagement in specific digital activities, such as social networking, information seeking, online shopping, and entertainment.

### Methodological quality

Table 1 presented the results of the methodological quality assessment for all 28 studies included in this systematic evaluation. Overall, the included studies demonstrated high methodological quality. Specifically, the mean AHRQ score for cross-sectional studies was 7.57, with 12 studies categorized as low risk of bias and 11 as moderate risk of bias. For cohort studies, the mean NOS score was 7.60, with 4 studies categorized as low risk of bias and 1 study categorized as moderate risk of bias. The most common sources of potential bias were the failure to report non-response rates and missing data handing procedures.

### Analysis of continuous outcomes

#### Main effects and heterogeneity

A total of 19 studies involving 27 effect sizes were included in the analysis of continuous outcomes. The three-level meta-analysis revealed a significant negative association between digital smart device use and depression scores. The pooled *β* was −0.201 (95% *CI*: −0.324 to −0.079, *p* < 0.01), indicating that digital device use is associated with lower levels of depression (Table 2).

**Table 2:** Results of the random effects model for the association between digital smart device use and depression

Outcome	Studies	*k*	Pooled_ES	95% *CI*	*p*	95% *PI*	Level 1 (%)	Level 2 variance Level 2 (%)	Level 3 variance Level 3 (%)
*β*	19	27	−0.201	[−0.324, −0.079]	< 0.01	[−0.675, 0.273]	0.87	0.002^**^ 3.61	0.053^***^ 95.52
OR	7	17	0.676	[0.613, 0.745]	< 0.001	[0.515, 0.886]	52.66	0.017 47.34	0 0

*k* number of effect sizes, *Pooled_ES* mean effect size, 95% *CI* = 95% confidence interval, 95% *PI* = 95% prediction interval, Level 1 (%) = sampling variance of observed effect sizes; Level 2 variance = variance between effect sizes extracted from the same study; Level 3variance = variance between studies. Asterisks indicate the statistical significance of variance components based on Likelihood Ratio Tests (LRT): *p < 0.05, **p < 0.01, ***p < 0.001

Variance decomposition revealed that the majority of the heterogeneity was attributable to between-study differences (level 3), accounting for 95.52% of the total variance. The estimated variance for this level was statistically significant (*σ*^*2*^ = 0.053, *p* < 0.001). In contrast, within-study variance (level 2) accounted for only 3.61% of the total heterogeneity, though it was also statistically significant (*σ*^*2*^ = 0.002, *p* < 0.01). Sampling variance (level 1) contributed the remaining 0.87%. These results indicate that the variation in effect sizes is primarily driven by differences across studies rather than within studies.

#### Moderator analysis

Univariate three-level meta-regression analyses were conducted to explore potential sources of heterogeneity (Table 3). The results indicated that none of the examined moderators significantly modified the association between digital device use and depression (all *p* for interaction > 0.05). This suggests that the protective effect is consistent across different demographic and study characteristics.

**Table 3:** Results of moderators for the association between digital smart device use and depression (continuous outcome)

Moderator	*k*	*β* (95% *CI*)	*F*	*p*	Level 2 variance	Level 3 variance	
Age							
≤ 70	14	−0.193 [−0.369, −0.017]^*^	0.056	0.814	0.002^**^	0.059^**^	
> 70	13	−0.224 [−0.412, −0.036]^*^					
Study design							
CS	23	−0.202 [−0.345, −0.059]^**^	0.030	0.863	0.002^**^	0.059^***^	
Cohort	4	−0.231 [−0.526, 0.064]					
Sample size							
≤ 8000	15	−0.115 [−0.252, 0.022]	2.487	0.092	0.002^**^	0.040^**^	
> 8000	12	−0.308 [−0.485, −0.130]^***^					
Region							
China	22	−0.221 [−0.375, −0.068]^**^	0.072	0.789	0.002^**^	0.062^***^	
Other regions	5	−0.180 [−0.439, 0.079]					
Depression measurement							
CES-D	_22_	−0.260 [−0.398, −0.121]^***^	2.775	0.096	0.002^**^		0.046^**^
Others	_5_	−0.046 [−0.256, 0.165]					
Publication year							
≤ 2020	7	−0.277 [−0.515, −0.040]^*^	0.476	0.490	0.002^**^		0.058^***^
> 2020	20	−0.178 [−0.330, −0.026]^*^					

*k* = number of effect sizes; 95% *CI* = 95% confidence interval; Level 2 variance = variance between effect sizes extracted from the same study; Level 3variance = variance between studies; For study design: CS = cross-sectional study; For region: Other regions including America, Japan and Korea; For depression measurement: Others including GDS, DTS and PHQ-2

^*^p < 0.05, **p < 0.01, ***p < 0.001

#### Sensitivity analysis and publication bias

The leave-one-study-out sensitivity analysis confirmed the robustness of the results. The overall pooled effect size was −0.201 under the three-level random-effects model. After sequentially excluding each study, the effect size fluctuated between −0.169 and −0.223 with minimal deviation from the overall estimate, indicating a high degree of robustness.

**Fig. 2:**
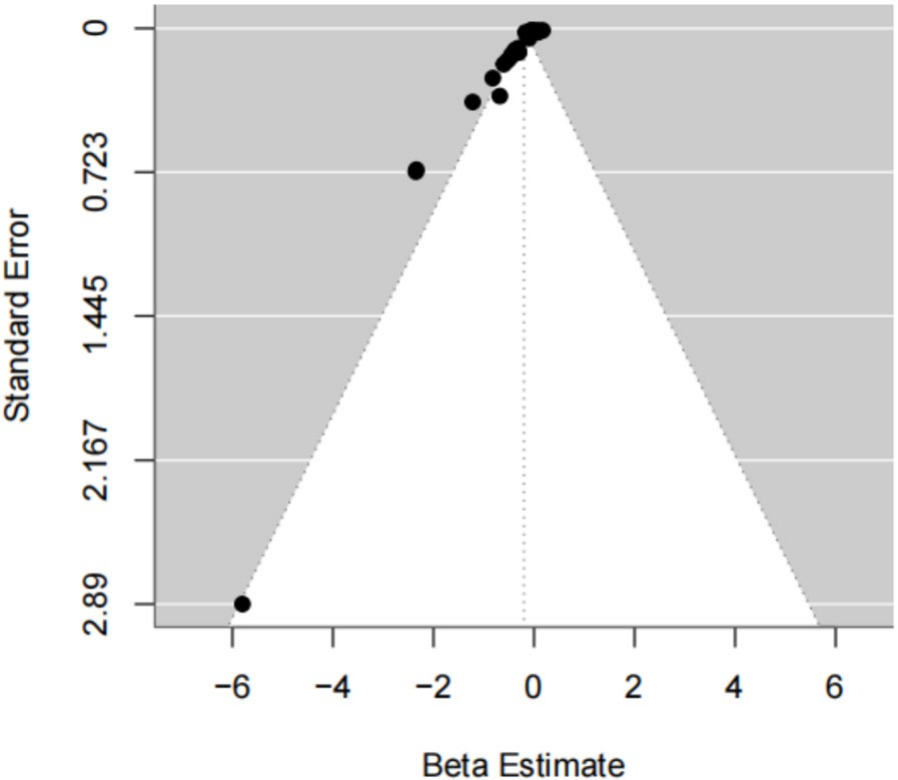
Funnel plot between digital smart device use and depression (continuous outcome)

The funnel plot indicated noticeable asymmetry (Fig. 2) and the modified Egger’s regression test further confirmed significant small-study effects (*p* < 0.001). To address this, a trim-and-fill analysis was performed. The analysis imputed 8 potentially missing studies, yet the adjusted pooled effect size remained statistically significant (*β* = −0.141, 95% *CI*: −0.269 to −0.013, *p* < 0.05). These results suggest that despite the presence of publication bias, the association between digital device use and lower depression scores remains robust.

### Analysis of binary outcomes

#### Main effects and heterogeneity

For binary outcomes, 7 studies providing 17 effect sizes were synthesized. The pooled OR was 0.676 (95% *CI*: 0.613 to 0.745, *p* < 0.001), suggesting that older adults using digital smart devices had a 32.4% lower risk of depression compared to non-users (Table 2).

Variance decomposition showed that 52.66% of the total variance was attributable to sampling error (level 1) and 47.34% to within-study heterogeneity (level 2, *σ*^*2*^ = 0.017, *p* > 0.05). The between-study variance (level 3) was negligible (0%). Crucially, the variance components at both level 2 and level 3 were not statistically significant, indicating high consistency in the protective effect of digital smart device use across different studies and within-study subgroups.

#### Moderator analysis

Univariate three-level meta-regression analyses were performed to identify potential moderators (Table 4). Similar to the continuous outcomes, no significant moderators were identified. The association remained stable across different age groups, study designs, sample sizes, regions, measurement tools, and publication years (all *p* > 0.05), reinforcing the generalizability of the findings.

**Table 4:** Results of moderators for the association between digital smart device use and depression (binary outcome)

Moderator	*k*	OR (95% *CI*)	*F*	*p*	Level 2 variance	Level 3 variance	
Age							
≤ 70	9	0.709 [0.593, 0.846]^***^	0.197	0.658	0.007	0.014	
> 70	8	0.668 [0.551, 0.810]^***^					
Study design							
CS	15	0.685 [0.601, 0.780]^***^	0.002	0.964	0.011	0.008	
Cohort	2	0.680 [0.507, 0.912]^*^					
Sample size							
≤ 8000	7	0.722 [0.618, 0.844]^***^	0.849	0.357	0.010	0.007	
> 8000	10	0.649 [0.549, 0.766]^***^					
Region							
China	10	0.693 [0.570, 0.843]^***^	0.012	0.994	0.006	0.022	
Japan	2	0.680 [0.493, 0.939]^*^					
Korea	5	0.684 [0.497, 0.941]^*^					
Depression measurement							
CES-D	9	0.710 [0.581, 0.868]^***^	0.238	0.888	0.006		0.022
GDS	3	0.650 [0.485, 0.872]^**^					
SGDS-K	_5_	0.685 [0.499, 0.941]^*^					
Publication year							
≤ 2020	8	0.709 [0.590, 0.851]^***^	0.175	0.675	0.008		0.012
> 2020	9	0.671 [0.563, 0.800]^***^					

*k* = number of effect sizes; 95% *CI* = 95% confidence interval; Level 2 variance = variance between effect sizes extracted from the same study; Level 3variance = variance between studies

#### Sensitivity analysis and publication bias

Sensitivity analyses demonstrated that the pooled results were stable. The overall pooled OR was 0.676. Excluding individual studies resulted in minor variations, with the pooled OR ranging from 0.690 to 0.698, indicating that no single study disproportionately influenced the results.

**Fig. 3:**
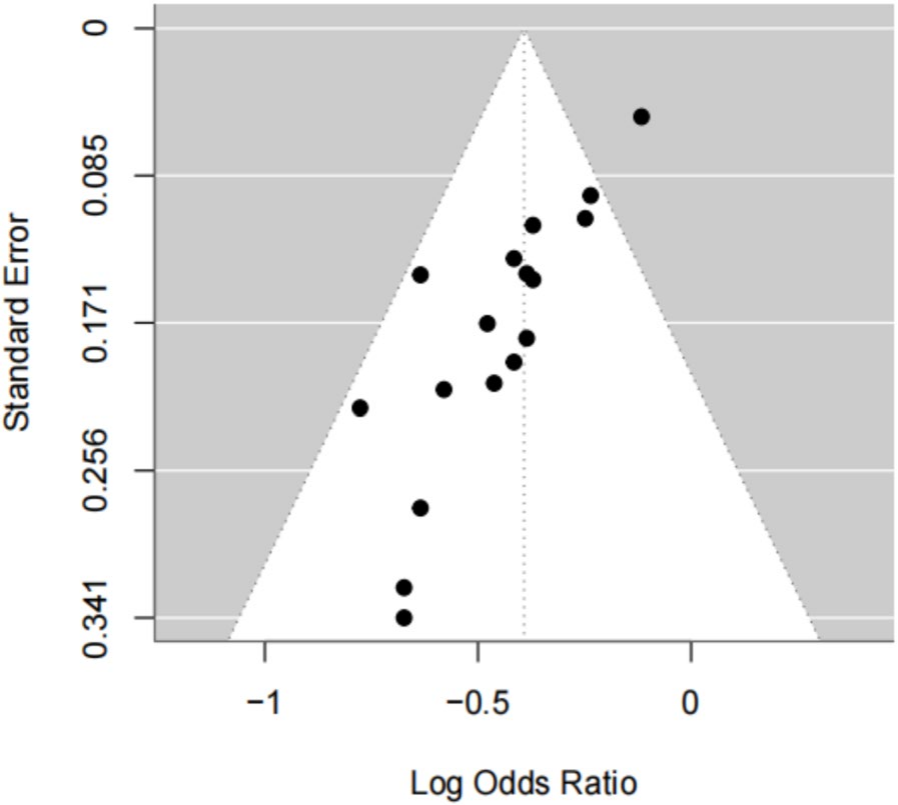
Funnel plot between digital smart device use and depression (binary outcome)

The funnel plot exhibited noticeable asymmetry (Fig. 3), and the modified Egger’s test indicated significant publication bias (*p* < 0.001). Consequently, a trim-and-fill analysis was conducted using the DerSimonian-Laird estimator. The analysis imputed 8 missing studies, but the adjusted pooled effect size remained highly significant (OR = 0.745, 95% *CI*: 0.677 to 0.819, *p* < 0.001). This confirms that the protective effect of digital device use against depression risk is genuine and not solely an artifact of publication bias.

### Narrative synthesis of the association between digital smart device use and depression among older adults

This study also summarized findings from four studies that reported *r* between digital smart device use and depression among older adults. The results were inconsistent, with two studies indicating a positive correlation, suggesting that digital smart device use was associated with higher depressive symptoms, potentially due to excessive screen time or reduced face-to-face interaction. In contrast, the other two studies found a negative correlation, implying that digital smart device use may be associated with lower depressive symptoms. The discrepancies could be attributed to variations in usage patterns, measurement approaches, or sample demographics. Further investigation is warranted to elucidate the underlying mechanisms and moderating factors influencing this association.

## Discussion

This three-level meta-analysis systematically synthesized existing evidence to explore the association between digital smart device use and depression among older adults. By modeling dependence among multiple effect sizes within studies and accounting for heterogeneity at different levels, our findings demonstrated a statistically significant protective association between digital smart device use and late-life depression across both continuous and binary outcomes. Although the observed effect size was small to moderate, it is comparable to other established behavioral determinants of mental health, such as physical activity, sleep quality, and social interaction [62–64]. Given the rising prevalence of both digital technology adoption and depression in aging populations [65, 66], these findings provide a robust empirical foundation for understanding the intersection of technology and geriatric mental health, as well as for the development of targeted interventions and policy initiatives.

The protective association observed in this study can be interpreted through the Selection, Optimization, and Compensation (SOC) model [67]. From this perspective, digital smart devices function as compensatory resources that help older adults adapt to age-related declines in mobility, sensory functioning, and social networks by enabling alternative pathways for communication, information access, and cognitive stimulation. Furthermore, Socioemotional Selectivity Theory (SST) offers additional explanatory power regarding the motivation behind device use [68]. As individuals age, they increasingly prioritize emotionally meaningful goals and relationships. Consequently, older adults may engage with digital technologies in more purposeful and instrumental ways, such as maintaining close relationships and accessing health information, rather than primarily for the status-seeking or social comparison behaviors often seen in younger cohorts [69]. Our findings extend these lifespan theories into the digital context, suggesting that technology use in later life should be conceptualized as an adaptive behavioral strategy.

Moderator analyses indicated that the association between digital smart device use and depression remained relatively stable across diverse demographic subgroups and cultural contexts. Nevertheless, the variance decomposition in our three-level model revealed distinct patterns of heterogeneity between the two types of outcomes. For continuous outcomes, the vast majority of variability resided at the between-study level, which highlights meaningful differences in the operationalization of digital smart device use across studies. These studies ranged from assessing general device ownership to measuring specific application engagement, suggesting that such conceptual breadth could impact the interpretability of results by grouping divergent digital behaviors under a single broad construct. In contrast, the between-study variance for binary outcomes was negligible, indicating a high degree of consistency in the protective effect when depression was treated as a categorical risk. This divergence implies that while the presence of a protective benefit is stable across different study populations, the degree of symptom reduction is more sensitive to how technology use is defined and measured. Therefore, future research must move beyond binary indicators to incorporate more standardized and nuanced assessments of digital engagement to fully elucidate these mechanisms.

The binary and continuous outcome analyses both identified significant publication bias. This suggests that studies with non-significant or negative findings may be underrepresented in the literature, potentially skewing the overall effect size [70, 71]. Notably, the majority of the included studies were conducted in East Asia, and this geographical concentration might influence the observed publication trends [72, 73]. While robustness checks using the trim-and-fill method confirmed the stability of the primary findings, the presence of bias underscores the necessity for comprehensive literature searches that include grey literature and unpublished studies. Researchers are encouraged to report all study results regardless of statistical significance to ensure a more complete and unbiased body of evidence.

From a practical standpoint, the statistically significant effect observed in this meta-analysis has profound implications for public health. Late-life depression is a complex condition influenced by multiple interacting biological, psychological, and social factors [74], meaning no single modifiable behavior is expected to exert a massive isolated effect. However, even modest protective associations can yield meaningful reductions in disease burden when applied across large populations. Therefore, digital smart device use should be recognized as a scalable, non-pharmacological component of geriatric care. At the individual level, interventions should emphasize purpose-driven and socially oriented uses of digital smart devices. Training programs must go beyond basic technical skills to help older adults adopt devices in ways that maximize social connectivity and cognitive benefits while minimizing potential risks like eye strain or sedentary behavior. At the community level, technology-supported social initiatives, including virtual interest groups and intergenerational digital mentoring programs, may promote meaningful engagement and reduce the digital divide. At the policy level, integrating digital tools into aging-in-place strategies and geriatric mental health services may offer a scalable and cost-effective complement to traditional prevention and intervention approaches. Importantly, digital smart devices should be viewed as an adjunct to established mental health care rather than as a replacement.

## Limitations

Several limitations of the meta-analysis should be considered when interpreting the results. First, the majority of the included studies employed cross-sectional designs, which may limit the ability to draw causal inferences. Future studies should prioritize longitudinal and experimental designs to examine the causal relationship between technology use and mental health outcomes. Second, substantial between-study heterogeneity remained for continuous outcomes despite employing a three-level meta analytic approach. Our moderator analyses did not identify demographic factors as significant sources of this variation, suggesting that the heterogeneity is likely attributable to unmeasured characteristics of digital smart device use. Future studies should quantify specific usage patterns to unpack this variability. Third, evidence of publication bias was detected, indicating that the true effect size might be more conservative than estimated. Future meta-analysis should strive to incorporate grey literature and unpublished data to minimize such bias. Finally, the geographical concentration of studies in specific regions may limit the generalizability of the findings. Expanding research to include diverse cultural and socioeconomic contexts will be essential to explore how local digital infrastructure and cultural norms shape the relationship between digital device use and geriatric depression.

## Conclusion

In conclusion, this three-level meta-analysis provides robust evidence that digital smart device use is associated with lower levels of depression symptoms among older adults. The findings highlight that technology adoption serves as a valuable adaptive strategy for promoting mental health in later life. While the association appears consistent across diverse subgroups, the substantial heterogeneity in continuous outcomes underscores the complexity of this relationship and the significant role played by how digital use is operationalized. Despite the presence of publication bias, the protective effect remains statistically robust, reinforcing the potential of digital smart devices as a scalable, non-pharmacological adjunct to traditional geriatric care. Future research should transition toward more nuanced measurements of digital engagement and longitudinal designs to further elucidate the causal mechanisms and long-term benefits of technology use on mental well-being in aging populations.

## Supplementary Information

Supplementary Material 1: PRISMA 2020 Checklist

Supplementary Material 2: Table S1 Keywords of three search aspects. Table S2 Search strategy for PubMed. Table S3 Search strategy for Cochrane Library. Table S4 Search strategy for Embase. Table S5 Search strategy for Web of Science. Table S6 Search strategy for MEDLINE. Table S7 Search strategy for PsycINFO. Table S8 List of excluded studies with reasons for exclusion (*n*=15). Table S9 Operationalization of digital smart device use.

## Data Availability

The data that support the findings of this study are openly available in PubMed, Cochrane Library, Embase, Web of Science, MEDLINE, and PsycINFO.

## Abbreviations

OR: Odds ratios
NOS: Newcastle–Ottawa Scale
AHRQ: Agency for Healthcare Research and Quality
CI: Confidence intervals
PROSPERO: International Prospective Register of Systematic Reviews
PRISMA: Preferred Reporting Items for Systematic Reviews and Meta-Analyses
MOOSE: Meta-analysis of Observational Studies in Epidemiology
MeSH: Medical Subject Headings
REML: Restricted maximum likelihood estimator
LRT: Likelihood ratio tests
GDS: Geriatric Depression Scale
CES-D: Center for Epidemiologic Studies Depression Scale
SGDS-K: Geriatric Depression Scale Short Form Korean Version
DTS: Depressive Tendency Scale
PHQ-2: Patient Health Questionnaire-2
CS: Cross-sectional study
NA: Not applicable
ES: Effect size
PI: Prediction interval
SOC: Selection, Optimization, and Compensation
SST: Socioemotional Selectivity Theory
