# Supplementary material for: Association between digital smart device use and depression among older adults: systematic review and meta-analysis
Source: BMC Public Health. 2026 May 9;26:2028. doi: 10.1186/s12889-026-27433-x (PMC13330471; doi:10.1186/s12889-026-27433-x)
Supplement: Supplementary file 2 — Supplementary Material 2: Table S1 Keywords of three search aspects. Table S2 Search strategy for PubMed. Table S3 Search strategy for Cochrane Library. Table S4 Search strategy for Embase. Table S5 Search strategy for Web of Science. Table S6 Search strategy for MEDLINE. Table S7 Search strategy for PsycINFO. Table S8 List of excluded studies with reasons for exclusion (n=15). Table S9 Operationalization of digital smart device use. [file 12889_2026_27433_MOESM2_ESM.docx]

**Table of contents**

**Table S1** Keywords of three search aspects 2

**Table S2** Search strategy for PubMed 3

**Table S3** Search strategy for Cochrane Library 4

**Table S4** Search strategy for Embase 5

**Table S5** Search strategy for Web of Science 6

**Table S6** Search strategy for MEDLINE 7

**Table S7** Search strategy for PsycINFO 8

**Table S8** List of excluded studies with reasons for exclusion (n=15) 9

**Table S9** Operationalization of digital smart device use 11

**Table S1** Keywords of three search aspects

| **Search Aspects** | **Keywords** |
| --- | --- |
| (a)Aged/Elderly | aged, elderly, aging, seniors, senescence, biological aging, aging biological, “aged, 80 and over”, oldest old, elders, elder, Elder*, geriatric, older, older adult, older adults, older people, older persons, older individuals, old, old population, old age, old adult, old adults, aging population, aging adults |
| (b)Smartphones/Digital devices | phone*, smartphone*, mobile phone, cell phone, cellular phone, smart phone, digital device, electronic device, cell phone use, mobile phone use, computer*, tablet computer, laptop |
| (c)Depression | depress*, depressed, depressive, depressive symptom, emotional depression, major depression, major depressive disorder, MDD, sadness |

**Table S2** Search strategy for PubMed

| Search number | Query |
| --- | --- |
| #1 | "Aged"[MeSH Terms] |
| #2 | "Aged"[Title/Abstract] OR "Elderly"[Title/Abstract] OR "Aging"[Title/Abstract] OR "Seniors"[Title/Abstract] OR "Senescence"[Title/Abstract] OR "biological aging"[Title/Abstract] OR "aging biological"[Title/Abstract] OR "aged 80 and over"[Title/Abstract] OR "oldest old"[Title/Abstract] OR "Elders"[Title/Abstract] OR "Elder"[Title/Abstract] OR "elder*"[Title/Abstract] OR "Geriatric"[Title/Abstract] OR "Older"[Title/Abstract] OR "Older Adult"[Title/Abstract] OR "Older Adults"[Title/Abstract] OR "Older People"[Title/Abstract] OR "Older Persons"[Title/Abstract] OR "Older Individuals"[Title/Abstract] OR "Old"[Title/Abstract] OR "Old Population"[Title/Abstract] OR "Old Age"[Title/Abstract] OR "Old Adult"[Title/Abstract] OR "Old Adults"[Title/Abstract] OR "Aging Population"[Title/Abstract] OR "Aging Adults"[Title/Abstract] |
| #3 | #1 OR #2 |
| #4 | "Smartphone"[MeSH Terms] OR "Cell Phone"[MeSH Terms] OR "Cell Phone Use"[MeSH Terms] OR "Computers"[MeSH Terms] OR "computers, handheld"[MeSH Terms] |
| #5 | "phone*"[Title/Abstract] OR "smartphone*"[Title/Abstract] OR "mobile phone"[Title/Abstract] OR "cell phone"[Title/Abstract] OR "cellular phone"[Title/Abstract] OR "smart phone"[Title/Abstract] OR "digital device"[Title/Abstract] OR "electronic device"[Title/Abstract] OR "cell phone use"[Title/Abstract] OR "mobile phone use"[Title/Abstract] OR "computer*"[Title/Abstract] OR "Tablet Computer"[Title/Abstract] OR "laptop"[Title/Abstract] |
| #6 | #4 OR #5 |
| #7 | "Depression"[MeSH Terms] OR "Depressive Disorder"[MeSH Terms] OR "Depressive Disorder, Major"[MeSH Terms]" |
| #8 | "depress*"[Title/Abstract] OR "Depressed"[Title/Abstract] OR "Depressive"[Title/Abstract] OR "Depressive Symptom"[Title/Abstract] OR "Emotional Depression"[Title/Abstract] OR "Major Depression"[Title/Abstract] OR "Major Depressive Disorder"[Title/Abstract] OR "MDD"[Title/Abstract] OR "Sadness"[Title/Abstract] |
| #9 | #7 OR #8 |
| #10 | #3 AND #6 AND #9 |

**Table S3** Search strategy for Cochrane Library

| Search number | Query |
| --- | --- |
| #1 | MeSH descriptor: [Aged] explode all trees |
| #2 | (Aged OR Elderly OR Aging OR Seniors OR Senescence OR "biological aging" OR "aging biological" OR "aged, 80 and over" OR "oldest old" OR Elders OR Elder OR Elder* OR Geriatric OR Older OR "Older Adult" OR "Older Adults" OR "Older People" OR "Older Persons" OR "Older Individuals" OR Old OR "Old Population" OR "Old Age" OR "Old Adult" OR "Old Adults" OR "Aging Population" OR "Aging Adults"):ti,ab,kw |
| #3 | #1 OR #2 |
| #4 | MeSH descriptor: [Smartphone] explode all trees |
| #5 | MeSH descriptor: [Cell Phone] explode all trees |
| #6 | MeSH descriptor: [Cell Phone Use] explode all trees |
| #7 | MeSH descriptor: [Computers] explode all trees |
| #8 | (phone* OR smartphone* OR "mobile phone" OR "cell phone" OR "cellular phone" OR "smart phone" OR "digital device" OR "electronic device" OR "cell phone use" OR "mobile phone use" OR computer* OR "Tablet Computer" OR laptop):ti,ab,kw |
| #9 | #4 OR #5 OR #6 OR #7 OR #8 |
| #10 | MeSH descriptor: [Depression] explode all trees |
| #11 | (Depress* OR Depressed OR Depressive OR "Depressive Symptom" OR "Emotional Depression" OR "Major Depression" OR "Major Depressive Disorder" OR MDD OR Sadness):ti,ab,kw |
| #12 | #10 OR #11 |
| #13 | #3 AND #9 AND #12 |

**Table S4** Search strategy for Embase

| Search number | Query |
| --- | --- |
| #1 | 'aged'/exp |
| #2 | (aged:ab,ti OR elderly:ab,ti OR aging:ab,ti OR seniors:ab,ti OR senescence:ab,ti OR 'biological aging':ab,ti OR 'aging biological':ab,ti OR 'aged, 80':ab,ti) AND over:ab,ti OR 'oldest old':ab,ti OR elders:ab,ti OR elder:ab,ti OR elder*:ab,ti OR geriatric:ab,ti OR older:ab,ti OR 'older adult':ab,ti OR 'older adults':ab,ti OR 'older people':ab,ti OR 'older persons':ab,ti OR 'older individuals':ab,ti OR old:ab,ti OR 'old population':ab,ti OR 'old age':ab,ti OR 'old adult':ab,ti OR 'old adults':ab,ti OR 'aging population':ab,ti OR 'aging adults':ab,ti |
| #3 | #1 OR #2 |
| #4 | 'smartphone'/exp OR 'mobile phone'/exp OR 'cell phone use'/exp OR 'computer'/exp |
| #5 | phone*:ab,ti OR smartphone*:ab,ti OR 'mobile phone':ab,ti OR 'cell phone':ab,ti OR 'cellular phone':ab,ti OR 'smart phone':ab,ti OR 'digital device':ab,ti OR 'electronic device':ab,ti OR 'cell phone use':ab,ti OR 'mobile phone use':ab,ti OR computer*:ab,ti OR 'tablet computer':ab,ti OR laptop:ab,ti |
| #6 | #4 OR #5 |
| #7 | 'depression'/exp |
| #8 | phone*:ab,ti OR smartphone*:ab,ti OR 'mobile phone':ab,ti OR 'cell phone':ab,ti OR 'cellular phone':ab,ti OR 'smart phone':ab,ti OR 'digital device':ab,ti OR 'electronic device':ab,ti OR 'cell phone use':ab,ti OR 'mobile phone use':ab,ti OR computer*:ab,ti OR 'tablet computer':ab,ti OR laptop:ab,ti |
| #9 | #7 OR #8 |
| #10 | #3 AND #6 AND #9 |

**Table S5** Search strategy for Web of Science

| Search number | Query |
| --- | --- |
| #1 | TS=(Aged OR Elderly OR Aging OR Seniors OR Senescence OR "biological aging" OR "aging biological" OR "aged, 80 and over" OR "oldest old" OR Elders OR Elder OR Elder* OR Geriatric OR Older OR "Older Adult" OR "Older Adults" OR "Older People" OR "Older Persons" OR "Older Individuals" OR Old OR "Old Population" OR "Old Age" OR "Old Adult" OR "Old Adults" OR "Aging Population" OR "Aging Adults") |
| #2 | TS=(phone* OR smartphone* OR "mobile phone" OR "cell phone" OR "cellular phone" OR "smart phone" OR "digital device" OR "electronic device" OR "cell phone use" OR "mobile phone use" OR computer* OR "Tablet Computer" OR laptop) |
| #3 | TS=(Depress* OR Depressed OR Depressive OR "Depressive Symptom" OR "Emotional Depression" OR "Major Depression" OR "Major Depressive Disorder" OR MDD OR Sadness) |
| #4 | #1 AND #2 AND #3 |

((TS=(Aged OR Elderly OR Aging OR Seniors OR Senescence OR "biological aging" OR "aging biological" OR "aged, 80 and over" OR "oldest old" OR Elders OR Elder OR Elder* OR Geriatric OR Older OR "Older Adult" OR "Older Adults" OR "Older People" OR "Older Persons" OR "Older Individuals" OR Old OR "Old Population" OR "Old Age" OR "Old Adult" OR "Old Adults" OR "Aging Population" OR "Aging Adults")) AND TS=(phone* OR smartphone* OR "mobile phone" OR "cell phone" OR "cellular phone" OR "smart phone" OR "digital device" OR "electronic device" OR "cell phone use" OR "mobile phone use" OR computer* OR "Tablet Computer" OR laptop)) AND TS=(Depress* OR Depressed OR Depressive OR "Depressive Symptom" OR "Emotional Depression" OR "Major Depression" OR "Major Depressive Disorder" OR MDD OR Sadness)

**Table S6** Search strategy for MIDLINE

| Search number | Query |
| --- | --- |
| #1 | TS=(Aged OR Elderly OR Aging OR Seniors OR Senescence OR "biological aging" OR "aging biological" OR "aged, 80 and over" OR "oldest old" OR Elders OR Elder OR Elder* OR Geriatric OR Older OR "Older Adult" OR "Older Adults" OR "Older People" OR "Older Persons" OR "Older Individuals" OR Old OR "Old Population" OR "Old Age" OR "Old Adult" OR "Old Adults" OR "Aging Population" OR "Aging Adults") |
| #2 | TS=(phone* OR smartphone* OR "mobile phone" OR "cell phone" OR "cellular phone" OR "smart phone" OR "digital device" OR "electronic device" OR "cell phone use" OR "mobile phone use" OR computer* OR "Tablet Computer" OR laptop) |
| #3 | TS=(Depress* OR Depressed OR Depressive OR "Depressive Symptom" OR "Emotional Depression" OR "Major Depression" OR "Major Depressive Disorder" OR MDD OR Sadness) |
| #4 | #1 AND #2 AND #3 |

((TS=(Aged OR Elderly OR Aging OR Seniors OR Senescence OR "biological aging" OR "aging biological" OR "aged, 80 and over" OR "oldest old" OR Elders OR Elder OR Elder* OR Geriatric OR Older OR "Older Adult" OR "Older Adults" OR "Older People" OR "Older Persons" OR "Older Individuals" OR Old OR "Old Population" OR "Old Age" OR "Old Adult" OR "Old Adults" OR "Aging Population" OR "Aging Adults")) AND TS=(phone* OR smartphone* OR "mobile phone" OR "cell phone" OR "cellular phone" OR "smart phone" OR "digital device" OR "electronic device" OR "cell phone use" OR "mobile phone use" OR computer* OR "Tablet Computer" OR laptop)) AND TS=(Depress* OR Depressed OR Depressive OR "Depressive Symptom" OR "Emotional Depression" OR "Major Depression" OR "Major Depressive Disorder" OR MDD OR Sadness)

| Search number | Query |
| --- | --- |
| #1 | SU (Aged OR Elderly OR Aging OR Seniors OR Senescence OR "biological aging" OR "aging biological" OR "aged, 80 and over" OR "oldest old" OR Elders OR Elder OR Elder* OR Geriatric OR Older OR "Older Adult" OR "Older Adults" OR "Older People" OR "Older Persons" OR "Older Individuals" OR Old OR "Old Population" OR "Old Age" OR "Old Adult" OR "Old Adults" OR "Aging Population" OR "Aging Adults") |
| #2 | SU (phone* OR smartphone* OR "mobile phone" OR "cell phone" OR "cellular phone" OR "smart phone" OR "digital device" OR "electronic device" OR "cell phone use" OR "mobile phone use" OR computer* OR "Tablet Computer" OR laptop) |
| #3 | SU (Depress* OR Depressed OR Depressive OR "Depressive Symptom" OR "Emotional Depression" OR "Major Depression" OR "Major Depressive Disorder" OR MDD OR Sadness) |
| #4 | #1 AND #2 AND #3 |

**Table S7** Search strategy for PsycINFO

SU (Aged OR Elderly OR Aging OR Seniors OR Senescence OR "biological aging" OR "aging biological" OR "aged, 80 and over" OR "oldest old" OR Elders OR Elder OR Elder* OR Geriatric OR Older OR "Older Adult" OR "Older Adults" OR "Older People" OR "Older Persons" OR "Older Individuals" OR Old OR "Old Population" OR "Old Age" OR "Old Adult" OR "Old Adults" OR "Aging Population" OR "Aging Adults") AND SU (phone* OR smartphone* OR "mobile phone" OR "cell phone" OR "cellular phone" OR "smart phone" OR "digital device" OR "electronic device" OR "cell phone use" OR "mobile phone use" OR computer* OR "Tablet Computer" OR laptop) AND SU (Depress* OR Depressed OR Depressive OR "Depressive Symptom" OR "Emotional Depression" OR "Major Depression" OR "Major Depressive Disorder" OR MDD OR Sadness)

**Table S8** List of excluded studies with reasons for exclusion (n=15)

| Number | Tittle | First Author | Year of publication | Exclusion Reasons |
| --- | --- | --- | --- | --- |
| 1 | Community-based case-control study of depression in older people. Cases and sub-cases from the MRC-ALPHA Study | Copeland JR | 1999 | Reviews, conferences, dissertations |
| 2 | Digital Information Technology Use, Self-Rated Health, and Depression: Population-Based Analysis of a Survey Study on Older Migrants | Kouvonen A | 2021 | Data cannot be extracted |
| 3 | The Effects of Smartphone Use on Life Satisfaction in Older Adults: The Mediating Role of Depressive Symptoms | Sagong H | 2022 | Reviews, conferences, dissertations |
| 4 | Internet accessibility and incident depressive symptoms in middle aged and older adults in China: A national longitudinal cohort study | Zhang H | 2023 | Wrong outcome |
| 5 | Investigating the links between objective social media use, attentional control, and psychological distress | Jones CN | 2024 | Wrong Population |
| 6 | Predictors of older adults' technology use and its relationship to depressive symptoms and well-being | Elliot AJ | 2014 | Data cannot be extracted |
| 7 | Purposes of Internet Use and Its Impacts on Physical and Psychological Health of Korean Older Adults | Jeon GS | 2024 | Wrong Population |
| 8 | Smartphone Use, Digital Addiction and Physical and Mental Health in Community-dwelling Older Adults: a Population-based Survey | Bertocchi FM | 2022 | Data cannot be extracted |
| 9 | Use of technology to enhance mental health for older adults | Cangelosi PR | 2014 | Reviews, Conferences, Dissertations |
| 10 | Variety, frequency, and type of Internet use and its association with risk of depression in middle- and older-aged Chinese: A cross-sectional study | Liao S | 2020 | Wrong Population |
| 11 | Fear of COVID-19, prolonged smartphone use, sleep disturbances, and depression in the time of COVID-19: A nation-wide survey | Li G | 2022 | Wrong Population |
| **Table S8: Continued** | | | | |
| Number | Tittle | First Author | Year of publication | Exclusion Reasons |
| 12 | Multidimensional Internet Use, Social Participation, and Depression Among Middle-Aged and Elderly Chinese Individuals: Nationwide Cross-Sectional Study | Du X | 2023 | Wrong Population |
| 13 | Internet paradox. A social technology that reduces social involvement and psychological well-being? | Kraut R | 1998 | Wrong Population |
| 14 | Person-Specific Analyses of Smartphone Use and Mental Health: Intensive Longitudinal Study | Cerit M | 2025 | Wrong Population |
| 15 | Tracking the Prevalence of Depression Among Older Adults in Singapore: Results From the Second Wave of the Well-Being of Singapore Elderly Study | P V A | 2025 | Reviews, Conferences, Dissertations |

**Table S9** Operationalization of digital smart device use

| **Study ID** | **Device type** | **Recall period** | **Operationalization** | **Specific activities** | **Duration/Frequency** |
| --- | --- | --- | --- | --- | --- |
| Uemura | Computers | past month | Binary (Yes/No) | - | - |
| Nakagomi | Smartphones Computers Tablets | past year | Binary (Yes/No) | Communication with friends/family  Social media  Information collection  Searching for medical facilities  Purchase of drugs/vitamins  Shopping  Banking | - |
| Lin | Smartphones Computers Tablets | - | Binary (Yes/No) | Social  Entertainment  Information | - |
| Zhang | Smartphones Computers Tablets | past month | Binary (Yes/No) | - | Never Not regularly(A few times a week)  Regularly(Almost every week/day) |
| Hwang | Smartphones Computers Tablets | - | Binary (Yes/No) | Send and receive text messages  Search for information  Take photos or videos  Listen to music  Play games  Watch videos  Use social networking services  Shop online  Conduct online financial transactions  Search for and install applications | - |
| Wang | Computers | - | Binary (Yes/No) | - | - |
| Liao | Smartphones Computers Tablets | - | Categorical  (Device Count: 0, 1, ≥2) | - | Never  Very rarely  Almost every week/day |
| Minagawa | Smartphones | - | Binary (Yes/No) | - | - |
| Ma | Smartphones Computers Tablets | - | Binary (Yes/No) | Online chatting  Watching the news Watching videos  Playing games,  Financial management | - |
| Chen | Computers | - | Binary (Yes/No) | - | - |
| Ji | Smartphones | - | Binary (Yes/No) | - | - |
| **Table S9: Continued** | | | | | |
| **Study ID** | **Device type** | **Recall period** | **Operationalization** | **Specific activities** | **Duration/Frequency** |
| Lee | Smartphones Computers Tablets | - | Binary (Yes/No) | Receiving text-messages  Sending text-messages  Searching for information  Taking photos and video recording  Listening to music  Playing games  Watching videos  Using social networking services  Online shopping | - |
| Mu | Smartphones | past month | Binary (Yes/No) | - | - |
| Choudhary | Smartphones Computers Tablets | - | Binary (Yes/No) | - | Never  Not regularly Almost every week  Almost every day |
| Xie | Smartphones | - | Binary (Yes/No) | - | - |
| Lu | Smartphones | past month | Binary (Yes/No) | - | - |
| Cotten | Smartphones Computers Tablets | - | Binary (Yes/No) | - | - |
| Liu | Smartphones Computers Tablets | - | Binary (Yes/No) | - | Average daily usage time |
| Ding | Smartphones | - | Binary (Yes/No) | Call  Text  Take photos  Check the weather  Social media  Games  E-books | Daily usage time:  <1 hour  1–3 hours  3–5 hours  ≥5 hours |
| Kim | Smartphones Computers Tablets | past month | Binary (Yes/No) | Health  Finance  Shopping | Rarely  Sometimes  Frequently |
| Guo | Smartphones Computers Tablets | past month | Binary (Yes/No) | - | - |
| Jiao | Smartphones Computers Tablets | - | Binary (Yes/No) | Interpersonal communication  Leisure and entertainment  Information acquisition | Low frequency  Medium frequency  High frequency |
| Guo | Smartphones Computers Tablets | - | Binary (Yes/No) | - | - |
| **Table S9: Continued** | | | | | |
| **Study ID** | **Device type** | **Recall Period** | **Operationalization** | **Specific activities** | **Duration/Frequency** |
| Nan | Computers | - | Binary (Yes/No) | Chat  Watch short videos  Shop online  Play online games  Learn online | - |
| Karaş | Smartphone | - | Binary (Yes/No) | - | Daily usage time:  1–3 hours  3–5 hours  ≥5 hours Durable years：  <1 year  1–3 years  3–6 years  ≥7 years |
| Chopik | Smartphones | - | Binary (Yes/No) | - | - |
| Koong | Smartphones Computers Tablets | - | Binary (Yes/No) | Receive messages  Send messages  Search and query  Take photos or record videos  Listen to music  Play games  Watch videos  Social networking services  E-commerce  Financial transactions  Search and install applications | - |
| Tian | Smartphones Computers Tablets | - | Binary (Yes/No) | - | - |

*Note*:-, not reported
